# Bioelectrical impedance outperforms waist circumference for predicting cardiometabolic risk in Congolese hypertensive subjects: a cross-sectional study

**DOI:** 10.1186/s12872-015-0011-7

**Published:** 2015-03-10

**Authors:** Philippe Bianga Katchunga, Achile Mwira Bapolisi, Jean-René M’Buyamba-Kabangu, Michel P Hermans

**Affiliations:** Cardiology Unit, Department of Internal Medicine, Provincial General Hospital of Bukavu, Catholic University of Bukavu, Bukavu, The Democratic Republic of Congo; Hypertension Unit, Department of Internal Medicine, University of Kinshasa Hospital, Kinshasa, The Democratic Republic of Congo; Department of Endocrinology, Saint Luc Academic Hospital, Brussels, Belgium

**Keywords:** Bioelectrical impedance, Waist circumference, Hypertensive, Congolese

## Abstract

**Background:**

Waist circumference threshold values used in sub-Saharan Africa correspond to those of European populations and are therefore inappropriate. Thus, they may over predict insulin resistance, especially in hypertensive Africans, in whom there is often no association between blood pressure and insulin resistance. Using bioelectrical impedance measurement in sub-Saharan Africa could possibly be advantageous to overcome the shortcomings of waist circumference measurement. The aim of this study was to evaluate the contribution of body composition estimation by bioelectrical impedance to predict cardiometabolic risk in Congolese hypertensive subjects.

**Methods:**

Cardiovascular profiling and body composition analysis by bioelectrical impedance was measured in 400 patients (men = 40%; age = 51.1 ± 12.6 years). Patients were diagnosed with a metabolic syndrome (MS) according to the IDF Criteria with and without the “blood pressure” criterion to remove any confounding autocorrelation bias, a visceral fat-MS (with and without the “blood pressure” criterion) being defined by the presence of ≥ 2 criteria with the precondition of excess visceral fat defined by a bio impedance measurement score >10/30. Total cardiovascular risk was assessed using the criteria of Framingham-2008.

**Results:**

The frequencies of enlarged waist circumference (71.9% vs 68.9%, p = 0.52) and IDF-MS without blood pressure criterion (24.9% vs 21.9%, p = 0.48) were similar among hypertensive vs. non hypertensive however excess visceral fat (57.6% vs 33.8%, p <0.0001) as well as visceral fat-MS without blood pressure criterion (18.9% vs 11.3%, p = 0.04) were more prevalent among hypertensive. Finally, total cardiovascular risk as well as arterial hypertension risk were associated with visceral fat, but not with waist circumference (p > 0.05).

**Conclusions:**

Pending the determination of thresholds values for pathological waist circumference adapted to sub-Saharan populations, using bioelectrical impedance measurement may contribute to better characterize the cardiometabolic risk and the insulin resistant phenotype of hypertensive sub-Saharan Africans.

## Background

Arterial hypertension (AHT) is a global public health problem affecting more than one billion individuals [1]. This worldwide increase in prevalence is conducted mainly by increased life expectancy [2] and by the epidemic of obesity and metabolic syndrome (MS) [3]. The mechanisms underlying the association between AHT and obesity are complex, with a major contribution from insulin resistance and compensatory hyperinsulinaemia [4]. The latter is associated with whole-body and visceral fat (VF), chronic low-grade systemic inflammation, abnormal glucose homeostasis, dyslipidemia, and elevated blood pressure [5], contributing to the MS phenotype [6]. The MS increases cardiovascular morbidity and mortality regardless of gender [7,8]. In hypertensive subjects, the presence of a MS predicts higher total cardiovascular (CV) risk and requires early and effective antihypertensive therapy [9]. Screening for MS in hypertensive patients may help identify a subset of patients requiring stricter cardiovascular and cardio metabolic prevention.

The current 5-items screening criteria for the MS include enlarged waist circumference (WC) next to elevated blood pressure (BP) and three biological parameters (hyperglycemia, hypertriglyceridemia, and hypo-HDL-cholesterolemia) [10], on which each one is easily obtainable in order to facilitate diagnosis.

In sub-Saharan Africa, AHT is highly prevalent in its population [11]. However, numerous studies reported a lack of association between BP and insulin resistance in the black population of this region [12].

On the other hand, there are currently few reliable regional data from sub-Saharan Africa allowing to establish consensual thresholds values for enlarged WC to be applied to the general population [10] Thus, current guidelines recommend using threshold values as defined for European populations to define enlarged WC in sub-Saharan Africans [10], regardless of ethnic or regional disparities in bio anthropometrics and central fat distribution. However, some studies show that the threshold values for pathological WC need to be adjusted to African people, especially for the female gender [13-17]. Thus, relying on WC threshold values currently recommended for sub-Saharan Africa remains inadequate and would certainly over predict insulin resistance, especially in hypertensive subjects.

In addition, measurement of WC is operator-dependent and prones to confounders, such as the respiratory cycle and the postprandial state [18]. Moreover, this measure does not discern subcutaneous from excess VF. [18]. Given these limitations, bioelectrical impedance is a simple, non-invasive and inexpensive means to qualitatively estimate VF [19], previously validated against an unbiased method to estimate body composition.

Using bioelectrical impedance in sub-Saharan African hypertensive patients could possibly be advantageous to overcome the shortcomings of WC measurement. The present work has assessed whether body composition analysis by bioelectrical impedance outperforms WC measurement to predict cardiometabolic risk in hypertensive outpatients from South Kivu followed in a cardiology department.

## Methods

### Study population

This cross-sectional study included patients of Black ancestry, aged ≥20 years old and living in the province of South Kivu (Democratic Republic of Congo), who attended the clinic of general cardiology of the Provincial General Hospital of Bukavu. The inclusion period ran from June 1st, 2010 to June 1^st^, 2012. Pregnant women, subjects with edema, and bedridden patients were excluded from this study.

The study was approved by the Ethics Committee of the School of Medicine at catholic university of Bukavu. Written informed consent for participation in the study was obtained from participants.

### Clinical measurements

For each patient, the medical history of AHT, diabetes mellitus (DM) and tobacco smoking were investigated.

Then, each subject was placed still standing, size and stature being measured with a meter stick, and WC with a flexible tape at the end of mild expiration, between the lower rib margins and the iliac crest. Thereafter, weight, visceral fat, total body fat, skeletal muscle mass and body mass index were measured using the OMRON BF510® body composition monitor, which is fitted with eight applied sensors in hands and feet for accurate measurement of whole-body electrical impedance. In 66 normal subjects without a MS and with a Body Mass Index (BMI) <27.5 kg.m-2 (without significant obesity or overweight), average visceral fat ±2SD amounted to 10.6/30, with 95th percentile at 9.0/30, justifying the choice of >10/30 to define excess visceral fat in the present study. The same threshold value was also recommended by the manufacturer.

Then, BP was measured using an electronic device (Spengler TB-101) in a sitting position after the participants had remained seated for 5 minutes. The measurements were taken twice, 2 min apart. The average of the two consecutive measurements was retained for analysis.

At the end of the consultation, the patient was sent to the laboratory for venous blood puncture on the forearm in order to measure fasting glycaemia (with fasting period > 8 hours), cholesterol fractions and triglycerides.

A diagnosis of AHT was made when BP ≥ 140/90 mm Hg and/or in the presence of a current hypotensive treatment [9], diabetes mellitus (DM) was considered when fasting glycaemia was ≥ 126 mg/dl on two occasions [20] or when a previous history of DM was present, and obesity was defined as BMI ≥30 kg/m^2^, respectively [21]. The presence of a MS was defined according to the criteria proposed by the International Diabetes Federation (IDF), which include, as prerequisite, a WC ≥80 cm and ≥94 cm for women and men, respectively [21], in association with two or more of the following 4 criteria: fasting glycaemia ≥100 mg/dl or DM; BP ≥130/85 mmHg or AHT; HDL-cholesterol levels <50 mg/dl and <40 mg/dl, respectively, in women and men; and fasting triglycerides ≥ 150 mg/dl in both genders. Patients were also analyzed according to the presence of an IDF-MS without the BP criterion (including patients with scores ≥ 3/4) to remove any confounding autocorrelation bias. In this study, a VF-MS phenotype (with and without blood pressure criterion) was considered in the presence of at least two MS criteria excepted WC combined with excess VF, defined as a bio impedance score ≥ 10/30. Total cardiovascular (CV) risk was also assessed using the criteria of Framingham-2008 [22].

### Statistical analyses

Data from participants were processed using the Epi INFO® 2000 version 3.5.3 and the 12.4.0 MedCalc® softwares. Data are described, as frequencies or means ± 1 standard deviation or median (95% CI), when appropriate. The distribution of the variables was tested for normality using the Kolmogorov-Smirnov test. We used 2 ways and 1 way analysis of variance (ANOVA) and Newman-Keuls tests to compare means for normal distribution otherwise Kruskal-Wallis test were used, and the chi-square test for comparison of proportions. The association between quantitative variables was modeled using general linear regression stepwise method and that between qualitative variables using logistic regression. A p value <0.05 was considered for statistical significance.

## Results

### General Characteristics of the study population

The Table 1 presents the general characteristics of the study population. In total, four hundred (400) patients were included in the study. Average values were: 51.1 ± 12.6 years for age; 27.9 ± 5.3 kg/m^2^ for BMI; 95.0 ± 13.1 cm for WC; 9.8/30 ± 4.0/30 for VF.

**Table 1:** **General characteristics of the study population**

	**All patients**	**Men**	**Women**	**p**	**Patients with AHT**	**Patients without AHT**	**P**
	**n = 400 (100)**	**n = 160 (40.0)**	**n = 240 (60.0)**		**n = 249 (62.2)**	**n = 151 (37.8)**	
Men n (%)	160 (40.0)	-	-		99 (39.8)	61 (40.3)	0.87
Age (years)	51.1 (12.6)	53.4 (13.5)	49.6 (11.8)	0.003	55.0 (11.1)	44.6 (12.4)	<0.0001
SBP (mmHg)	146.0 (24.8)	148.5 (23.5)	144.3 (25.5)	0.09	154.6 (21.4)	119.0 (11.8)	<0.0001
DBP (mmHg)	87.3 (15.2)	90.1 (15.3)	85.6 (14.9)	0.004	91.5 (14.7)	74.4 (7.9)	<0.0001
BMI (Kg/m^2^)	27.9 (5.3)	25.8 (3.9)	29.2 (5.7)	<0.0001	28.1 (5.0)	27.4 (5.6)	0.26
WC (cm)	95.0 (13.1)	93.7 (12.1)	95.9 (13.7)	0.09	95.7 (13.0)	94.0 (13.1)	0.08
MM (%)	27.7 (5.8)	32.9 (4.7)	24.3 (3.5)	<0.0001	27.5 (5.3)	28.2 (6.5)	0.23
VF (0/30-30/30)	9.8 (4.0)	10.8 (4.7)	9.1 (3.3)	<0.0001	10.5 (4.1)	8.6 (3.4)	<0.0001
BF (%)	36.1 (11.6)	25.9 (7.5)	42.9 (8.5)	<0.0001	36.4 (11.2)	35.5 (12.3)	0.48
Glycemia (mg/dl)	109.5 (48.4)	107.0 (41.7)	111.4 (53.0)	0.43	106.6 (47.1)	114.4 (50.3)	0.16
TC (mg/dl)	207.2 (56.9)	205.5 (50.3)	208.2 (60.8)	0.64	210.4 (56.9)	201.9 (56.6)	0.06
HDL-C (mg/dl)	46.3 (16.1)	44.4 (12.2)	47.6 (18.1)	0.055	46.6 (15.6)	46.0 (16.8)	0.21
LDL-C (mg/dl)	136.4 (49.2)	137.1 (47.0)	136.1 (50.5)	0.85	138.6 (50.5)	132.8 (46.8)	0.26
TG (mg/dl)	121.5 (69.9)	122.6 (83.7)	120.8 (59.5)	0.81	125.9 (75.9)	114.4 (58.4)	0.11
DM (%)	37.2	33.6	39.9	0.24	37.7	36.4	0.80
Obesity (%)	33.4	33.5	43.8	<0.0001	34.4	31.8	0.59
AHT (%)	62.3	61.9	62.5	0.89	100	0.0	-
WC ≥ 80 cm (W) / 94 cm (M)	70.8	47.2	86.3	<0.0001	71.9	68.9	0.52
Glycemia > 100 mg/dl or DM	42.2	40.3	43.5	0.51	43.1	40.7	0.62
HDL-C < 50 mg/dl (W) / < 40 mg/dl (M)	45.9	39.6	50.0	0.04	45.0	47.3	0.66
TG > 150 mg/dl	25.8	22.1	28.2	0.18	26.7	24.2	0.57
BP ≥ 130/85 mmHg or AHT	81.3	83.9	78.8	0.21	100.0	50.3	<0.0001
VF ≥ 10/30	48.6	60.6	41.5	0.0002	57.6	33.8	<0.0001
IDF-MS	47.4	30.4	58.3	<0.0001	53.3	38.3	0.003
IDF-MS without BP criteria	23.8	-	-	-	24.9	21.9	0.48
VF-MS	34.4	39.4	31.1	0.08	44.7	18.0	<0.0001
VF-MS without BP criteria	16.0	-	-	-	18.9	11.3	0.04
Framingham score (%)	13.4 (9.6)	17.4 (10.0)	10.8 (8.3)	<0.0001	16.4 (9.3)	8.7 (7.9)	<0.0001

AHT = arterial hypertension; BMI = body mass index; BP = blood pressure; SBP = systolic blood pressure; DBP = diastolic blood pressure; WC = waist circumference; VF = visceral fat; BF = body fat; MM = skeletal muscle mass; TC = total cholesterol ; HDL = high density lipoprotein; LDL = low density lipoprotein; TG = triglycerides; DM: diabetes mellitus.

Both frequency of obesity (43.8% vs. 33.5%; p < 0.0001) and large WC (86.3% vs. 47.2%; p < 0.0001) were high in women than in men respectively. However, men presented significantly higher VF (10.8 ± 4.7/30 vs. 9.1 ± 3.3/30; p < 0.0001) and diastolic BP (90.1 ± 15.3 mmHg vs. 85.6 ± 14.9 mmHg; p = 0.004) than women.

Compared to normotensive patients (37.8%), hypertensive patients (62.2%) had a significantly higher age (55.0 ± 11.1 years vs 44.6 ± 12.4 years; p <0.0001) and VF score (10.5/30 ± 4.1/30 vs 8.6/30 ± 3.4/30; p <0.0001), whereas values were not statistically different with respectively BMI, WC, and other biological parameters (p > 0.05) between groups.

### Total cardiovascular risk in the studied population

The Table 2 reports total CV risk of our sample. Total CV risk of the studied population was 13,4 ± 9.6%. Total CV risk was significantly higher in men than women (17.4 ± 10.0% vs 10.8 ± 8.3; p < 0.0001). Moreover, patients in the 4^th^ VF quartile had a total CV risk significantly higher than those in the 3^rd^ lower quartiles in total as well in men and women separately (p < 0.05).

**Table 2:** **Total cardiovascular risk on the patients studied**

	**Framingham score (%)**		
	**All patients**	**Men**	**Women**
	**13.4 (9.6)**	**17.4 (10.0)**	**10.8 (8.3)**
**BMI Quartile (Kg/m** ^**2**^ **) [median (95% CI)]**			
1^st^ quartile [22.0 (22.0 to 23.0)]	13.1 (10.2)	15.6 (11.0)	10.2 (8.4)
2^nd^ quartile [26.0 (26.0 to 26.0)]	15.2 (10.0)	18.7 (9.9)	10.8 (8.4)
3^rd^ quartile [29.0 (29.0 to 30.0)]	13.2 (8.9)	18.2 (8.6)	10.7 (8.1)
4^th^ quartile [34.0 (33.0 to 35.9)]	12.1 (9.0)	17.9 (9.8)	11.1 (8.5)
p	0.17	0.40	0.95
**WC Quartile (cm) [median (95% CI)]**			
1^st^ quartile [79.0 (77.7 to 81.0)]	11.6 (10.0)	14.7 (11.1)	9.3 (8.5)
2^nd^ quartile [91.0 (90.0 to 92.0)]	13.8 (9.2)	18.9 (9.4)	10.2 (7.1)
3^rd^ quartile [99.0 (98.6 to 100)]	14.1 (9.6)	18.2 (9.4)	11.1 (8.6)
4^th^ quartile [108.5 (108.0 to 110)]	14.3 (9.5)	18.8 (9.5)	12.4 (8.9)
p	0.17	0.18	0.20
**VF Quartile (/30) [median (95% CI)]**			
1^st^ quartile [6.0 (5.0 to 6.0)]	10.8 (9.4)	14.7 (10.7)	8.6 (7.6)
2^nd^ quartile [9.0 (8.0 to 9.0)]	10.1 (8.6)	15.0 (10.2)	8.7 (7.5)
3^rd^ quartile [11.0 (10.0 to 11.0)]	14.7 (8.8)	17.7 (9.6)	13.1 (8.0)
4^th^ quartile [15.0 (14.0 to 15.0)]	18.7 (9.3)^*^	19.7 (9.2)^*^	16.3 (9.1)^*^
p	<0.0001	0.04	<0.0001

^*^p < 0.05 compared to the 1^st^, 2^nd^ and the 3^rd^ quartiles.

However, the total CV risk was similar between quartiles of BMI and WC in men and women (p > 0.05).

In stepwise general linear regression (Table 3), total CV risk was associated to VF (β coefficient = 0.24; partial r = 0.15; p = 0.002) and age. WC and BMI were rejected by the model (p > 0.05).

**Table 3:** **Multivariate linear stepwise regression analysis of Waist circumference, visceral fat, systolic blood pressure, diastolic blood pressure and total cardiovascular risk respectively according to the alleged risk factors**

	**β coefficient**	**Standard error**	**Partial r**	**p**
**WC (cm)**				
BMI (Kg/m^2^)	1.44	0.09	0.59	<0.0001
VF (0/30)	0.94	0.13	0.33	<0.0001
not included in the model (a)				
**VF (0/30)**				
Age (years)	0.07	0.009	0.36	<0.0001
Gender F(1) / M (2)	3.14	0.25	0.52	<0.0001
BMI (Kg/m^2^)	0.46	0.03	0.54	<0.0001
WC (cm)	0.05	0.01	0.20	<0.0001
**SBP (mmHg)**				
Age (years)	0.66	0.09	0.32	<0.0001
VF (/30)	0.75	0.29	0.12	0.01
Not included in the model (b)				
**DBP (mmHg)**				
Gender F (1) / M (2)	3.62	1.54	0.11	0.01
VF (/30)	0.61	0.18	0.16	0.001
Not included in the model (c)				
**Total cardiovascular risk (Framingham score)**				
Age (years)	0.57	0.02	0.75	<0.0001
VF (/30)	0.24	0.07	0.15	0.002
Not included in the model (d)				

(a) Age, gender; (b) BMI, gender, WC, total cholesterol; (c) Age, WC, BMI, total cholesterol (d) WC, BMI.

### Metabolic syndrome parameters

Table 1 reports the frequencies of the various components of the MS. In total, the frequency was respectively 47.4% for IDF-MS and 34.4% for VF-MS (p = 0.0003). Compared to men, the frequencies were higher in women significantly for large WC (86.3% vs 47.2%; p < 0.0001) and IDF-MS (58.3% vs 30.4%; p < 0.0001). However, similar values of VF-MS were found in both sexes (Men vs women: 39.4% vs 31.1%; p = 0.08).

Compared to non-hypertensive participant, hypertensive ones had a frequency of IDF-MS significantly higher (53.0% vs 38.0%; p = 0.003). However, the frequency of IDF-MS was similar in the 2 groups when the BP criterion was excluded (24.9% vs 21.9%; p = 0.48). Moreover, VF-MS with BP criterion (44.7% vs 18.0%; p < 0.0001) or without BP criterion (18.9% vs 11.3%; p = 0.04) remained significantly higher in the group of hypertensive patient than in the group of non-hypertensive patient.

### Determinants of blood pressure

The results of the general linear regression using stepwise method of BP according to the alleged risk factors are listed in Table 3. There was a significant positive association between systolic BP and VF (β coefficient = 0.75 mmHg; p = 0.01), and between diastolic BP and VF (β coefficient = 0.61 mmHg; p = 0.001) after adjusting for confounders factors. WC was not included in the model.

### Determinants of arterial hypertension

Table 4 shows the odds ratio for AHT by DM and quartiles of age, BMI, WC and VF, respectively. The results of the multivariate logistic regression show that, compared to patients in the 1^st^ VF quartile, patients in the 4^th^ visceral fat quartile were 2.9 times more frequently hypertensive after adjusting for age, BMI and WC (p <0.05). By contrast, the difference was not statistically significant for WC (p > 0.05).

**Table 4:** **Multivariable-adjusted Odds ratios for arterial hypertension**

	**All patients Adjusted OR (95%CI)**	**Men Adjusted OR (95% CI)**	**Women Adjusted OR (95%CI)**
Age (years)			
1^st^ quartile	1	1	1
2^nd^ quartile	2.3 (1.2 to 4.2)*	1.2 (0.4 to 3.5)	3.7 (1.7 to 7.8)*
3^rd^ quartile	4.3 (2.2 to 8.5)*	2.0 (0.7 to 5.7)	7.9 (3.1 to 19.6)*
4^fh^ quartile	9.3 (4.4 to 19.6)*	4.3 (1.5 to 11.9)*	23.5 (7.1 to 77.9)*
BMI (Kg/m^2^)			
1^st^ quartile	1	1	-
2^nd^ quartile	1.9 (0.8 to 4.2)	1.6 (0.3 to 8.3)	-
3^rd^ quartile	2.1 (0.8 to 5.3)	3.3 (0.3 to 31.8)	-
4^fh^ quartile	2.0 (0.7 to 5.7)	2.1 (0.1 to 25.1)	-
WC (cm)			
1^st^ quartile	1	1	-
2^nd^ quartile	1.0 (0.5 to 2.1)	2.1 (0.1 to 25.1)	-
3^rd^ quartile	0.4 (0.1 to 1.0)	0.3 (0.07 to 1.5)	-
4^fh^ quartile	0.5 (0.1 to 1.4)	0.2 (0.03 to 2.1)	-
VF (/30)			
1^st^ quartile	1	1	1
2^nd^ quartile	0.8 (0.4 to 1.7)	1.1 (0.3 to 4.1)	0.7 (0.3 to 1.5)
3^rd^ quartile	1.4 (0.6 to 3.4)	1.8 (0.3 to 9.6)	1.2 (0.5 to 2.7)
4^fh^ quartile	2.9 (1.0 to 7.8)*	7.3 (0.9 to 55.6)	0.9 (0.2 to 3.3)
DM	-		

*p<0.05.

However, when stratified by sex, the association between AHT and VF became statistically meaningless (p = 0.055), age was the major determinant.

## Discussion

This analysis of four hundred indigenous patients from South Kivu attending the outpatient cardiology clinic of Bukavu Reference General Provincial Hospital shows that bioelectrical impedance provides better discrimination between hypertensive and normotensive patients as regards visceral fat accumulation compared to standard metabolic syndrome criteria, including waist circumference, which were similar in both groups. In addition, visceral fat accumulation, unlike waist circumference, was significantly associated with total cardiovascular risk and arterial hypertension risk.

These results demonstrate the usefulness of measuring body composition by bioelectrical impedance to grade visceral fat accumulation, a major driver of insulin resistance, in hypertensive patients. Previously, Aydin M et al. reported that visceral fat estimated by bioelectrical impedance was significantly associated with low-grade systemic inflammation in Turkish adults [23]. Bioelectrical impedance is a validated unbiased method to estimate body composition, and an OMRON monitor fitted with 8 sensors at hands and feet for accurate measurement of whole-body electrical impedance, identical to that used for this study, was previously shown to have excellent correlation with both magnetic resonance imaging (MRI) and dual X-ray absorptiometry (DEXA) for estimating fat mass (r = 96%) and visceral fat (r = 92%), respectively [19]. Bioelectrical impedance is also advantageous in being more accessible in terms of cost and implementation than MRI, more cumbersome in terms of hardware, cost and staff training [24].

Two relevant observations derived from the present study. The first is that body composition estimated by bioelectrical impedance showed a high frequency of visceral fat accumulation in hypertensive vs normotensive patients in a South-Kivu population. Moreover, although Saad MF et al. reported that the association between blood pressure and insulin resistance was limited to Caucasians and absent among blacks and PIMA Indians [12], the present observation suggests a role for excess visceral fat (and insulin resistance) in the pathogenesis of arterial hypertension in black subjects corroborating other authors [25]. We noted, however, that another study reported a lack of correlation between blood pressure and insulin resistance in diabetic patients [26]. This discrepancy may have arisen from the confounding effect of a high frequency of atypical diabetes mellitus (ie. without insulin resistance) among these diabetic patients. High blood pressure in that study was associated with other determinants, including age and chronic kidney disease [26].

The second observation is the lack of difference in the frequency of MS between hypertensive and normotensive patients when blood pressure criteria were removed from the definition of the MS. By contrast, substituting the WC criterion with that of “high visceral fat” measured by bioelectrical impedance in the definition of the MS (with or regardless the blood pressure criterion) allowed for separating hypertensive from non-hypertensive patients. In addition, visceral fat, but not WC, was associated with total cardiovascular risk. Taken together, these results suggest a clear relevance of measuring visceral fat (as proxy for insulin resistance) to better characterize cardio metabolic risk in these Congolese hypertensive patients. Indeed, several limitations of the current criteria of the MS are particularly relevant and applicable to South Kivutians. First, the “waist” criterion extrapolated from Caucasian populations is not suitable to sub-Saharan Africans. [10] In addition, innate hypotriglyceridemia is frequent black populations, both in indigenous groups and in longstanding expatriates (such as African-Americans) or in contemporary migrants. [27] Similarly, DM without insulin resistance is common in this region [20]. Finally, some authors reported a lack of association between blood pressure and insulin resistance in people from black ancestry [12]. Thus, defining the presence of a MS using conventional criteria would be partly done another common mechanism as age which increases the frequency of all parameters of the metabolic syndrome [28]. This is unlikely in the present study.

The present study shows that higher visceral fat determined by bioelectrical impedance is an easy means to document higher cardiometabolic risk, including excess risk in hypertensive vs. normotensive patients. The association between cardiometabolic risk and visceral fat in the present study could not be ascribed to a confounding effect of other standard drivers of insulin resistance, such as age, gender, BMI and WC.

An obvious limitation of this work lies in its transversal design, which precludes drawing causal inferences between WC, visceral fat and cardiovascular events or cardiometabolic morbidities’ incidence. Similarly, the reference method of planimetry CT or DEXA to measure visceral fat could not be used as a plain result of non-availability. Whereas the bioimpedance method used in this study merely provides with a semi-quantitative (0–30) scale to estimate visceral fat accretion, is was nevertheless highly correlated with gold standards methods, such as whole body magnetic resonance imaging (MRI) and dual energy X-ray absorptiometry (DEXA) in healthy normal-weight, overweight and obese adults, [19]. In addition, the inclusion of subjects at high cardiovascular risk may be biased by confounders of body weight or body composition, including drug-induced body weight or body composition changes as a result of diuretics and glucose-lowering therapies. Another potential bias may arise from the fact that the cutoff value for visceral fat used in this study was similar for men and women, and do not equate real-life gender-adjusted thresholds [29], and generalizing the present results to the whole population needs confirmatory studies. For all these limitations, it is likely that defining appropriate regional threshold values for WC that would predict insulin resistance will remain for long an ongoing debate. Our results suggest that a simple estimation of visceral fat with a bio impedance method may provide a useful alternative to grade cardiometabolic risk in black populations.

## Conclusions

This study shows that estimating body composition by electrical bio impedance provides a simple, more precise alternative to WC as surrogate to insulin resistance in hypertensive Congolese. Thus, pending the determination of consensual population-based threshold values for enlarged WC, the alternative use of bio-impedance methods seems appropriate.
